# N-3 fatty acid supplementation mediates lipid profile, including small dense LDL, when combined with statins: a randomized double blind placebo controlled trial

**DOI:** 10.1186/s12944-022-01686-y

**Published:** 2022-09-01

**Authors:** Gediz Dogay Us, Sohail Mushtaq

**Affiliations:** 1 University of Chester, Parkgate Road, Chester, CH1 4BJ UK; 2 NUTRIM School of Nutrition and Translational Research In Metabolism, Maastricht University, Maastricht, Netherlands; 3 University of Chester, Faculty of Medicine, Dentistry and Life Sciences, Parkgate Road, Chester, CH1 4BJ UK

**Keywords:** Cardiovascular risk, Atherosclerosis, Hyperlipidemia, Lipoprotein, Low-density lipoprotein cholesterol size, Omega 3 polyunsaturated fatty acids, Small dense low-density lipoprotein cholesterol

## Abstract

**Background:**

Epidemiological and clinical evidence suggests that high-dose intake of omega 3 fatty acids (n-3 FA) have a favorable role in altering serum triglycerides (TG) and non-high density lipoprotein cholesterol (non-HDL-C) when combined with statins in hyperlipidemic patients. Their efficacy in altering low-density lipoprotein cholesterol (LDL-C) particle size is yet to be established.

**Aim:**

This study evaluated the effects of supplementing 4 g/day Eicosapentaenoic acid (EPA) and Docosahexaenoic acid (DHA) on serum blood lipids, including small, dense LDL-C particle concentration, in hyperlipidemic patients receiving stable statin therapy.

**Methods:**

In this randomized, placebo-controlled, double-blind parallel group study, 44 patients on statin therapy for > 8 weeks with non-HDL-C concentrations above 130 mg/dL were randomized into two groups. For 8 weeks, together with their prescribed statin, the intervention group received 4 g/day EPA + DHA (3000 mg EPA + 1000 mg DHA in ethyl ester form) and the placebo group received 4 g/day olive oil (OO). Measurements of serum non-HDL-C, TG, total cholesterol (TC), high density lipoprotein cholesterol (HDL-C), LDL-C (including large - LDL I; intermediate - LDL II; and small - LDL III subclasses), very-low-density lipoprotein cholesterol (VLDL-C) concentration, were taken at baseline and post-intervention. Dietary intake was assessed with a weighed intake, 3-day food diary at week 4. Primary outcome measures were percent change in LDL III, non-HDL-C and LDL particle number.

**Results:**

At the end of treatment, the median percent change in serum LDL III concentration was significantly greater in the n-3 FA group plus atorvastatin compared to placebo (− 67.5% vs − 0%, respectively; *P* < 0.001). Supplementation with n-3 FA plus atorvastatin led to significant reductions in serum non-HDL-C (− 9.5% vs 4.7%, *P* < 0.01), TG (− 21.5% vs 6.2%, *P* < 0.001) and VLDL-C (− 36.9% vs 4.0%, *P* < 0.001) and TC (− 6.6% vs 2.1%, *P* < 0.001). Between the groups, no significant difference in percent change in the serum concentration of LDL-C, HDL-C, as well as in the LDL I and LDL II subclasses was observed.

**Conclusion:**

In this group of hyperlipidemic patients on a stable statin prescription, OM3 plus atorvastatin improved small dense LDL concentrations, non-HDL-C, VLDL-C and TG to a greater extent than atorvastatin alone. Further studies are warranted in this area.

**Trial registration:**

This trial was retrospectively registered on 23 May 2019 on ClinicalTrials.gov with ID: NCT03961763.

## Background

Hyperlipidemia is one of the known etiological causes of atherosclerosis. Epidemiological studies have shown that elevated serum concentrations of TG and LDL-C as well as low concentrations of HDL-C are independently and partially linked to the development of atherosclerosis [1]. Although much focus has been on LDL-C as the primary therapeutic target of atherosclerosis, The National Lipid Association suggest that non-HDL-C might be a superior indicator of cardiovascular disease (CVD) risk than LDL-C is, based on clinical evidence [2]. More importantly, LDL-C particle size is also suggested to be an important determinant of atherosclerotic process. Small and dense LDL-C particles have an increased capacity to penetrate the arterial wall and are more readily oxidized compared to the large and buoyant ones [3]. Consistent with this, clinical studies collectively show a positive relation between serum concentration of small, dense LDL-C particles and CVD risk [4–10]..

Marine-derived n-3 FA EPA and DHA are known to play a role in altering lipid metabolism by upregulating FA catabolism through the nuclear receptor peroxisome proliferator–activated receptor-α (PPAR- α) activation and decreasing lipogenesis by downregulating sterol responsive element binding protein-1c (SREBP1-c) [11]. Although statins are the first drug of choice to alter blood lipids, accumulating evidence suggests that marine-derived n-3 FA EPA and DHA supplementation effectively decreased serum triglycerides and non-HDL-C in several clinical studies. In two large clinical trials EVOLVE (*n* = 399) and MARINE (*n* = 229), 4 g/day EPA and DHA supplementation alone resulted in 31%(*p* < 0.001) and 33% (*p* < 0.0001) reduction in serum TG concentrations as well as 10% (*p* < 0.01) and 18% (*p* < 0.0001) reduction in non-HDL-C concentrations, respectively [12, 13].. LDL-C, however, is usually reported to rise on EPA and DHA treatment, possibly due to increased production of LDL-C from VLDL-C [14]. In a systematic review, a 6 mg/dL (+ 3, + 8, *P* = 0.0006) increase in serum LDL-C concentrations was reported [15]. In the EVOLVE trial, LDL-C increased 19% (*p* < 0.001) with 4 g/day EPA and DHA intake [12]. However, the increase in LDL-C with EPA and DHA monotherapy is usually counter balanced when these fatty acids are combined with statins [16]. Indeed, clinical trials that compare statin monotherapy with n-3 combination therapies collectively show added benefits on blood lipid profile and conclude that EPA and DHA enhance statin-mediated improvements, with 30-55% reductions in TG, as well as 10-20% reductions in non-HDL-C without a concomitant increase in LDL-C [17–20]. In the COMBOS trial (*n* = 254), 4 g/day EPA and DHA supplementation combined with simvastatin resulted in 10% reduction in non-HDL-C (*p* < .001) with no accompanying increase in LDL-C [17], while the same study design in ESPRIT trial (*n* = 647) showed a 7% decrease in non-HDL-C (*p* < 0.01) with no significant change in LDL-C [18].

As opposed to the great number of clinical studies that investigate the role of n-3 FA in altering blood lipids, their effect on LDL-C particle size and concentration has not been widely investigated. A decrease in LDL III concentration from baseline (− 1.23 mmol/L ± 2.99, *p* < .05) was reported when 1.68 g/d EPA and DHA is combined with statin for 5 weeks, despite the relatively low-dose of n-3 FA supplements and short duration [21]. Similar results are reported with 4 g/day EPA and DHA combination therapy with statin [22]. Subsequent studies that tested the efficacy of EPA and DHA with statin and reported positive results, did not measure LDL-C particle size and concentration in their studies despite these earlier findings [18–20, 23, 24]. In another study, coadministration of n-3 FA with statin increased LDL particle size and decreased TG level in 51 dyslipidemic patients [25]. More recently, an improvement in atherogenic lipoprotein particle size and concentration when statin-prescribed patients were administered with 4 g/day EPA and DHA was reported, while TC, TG and LDL-C concentrations also significantly decreased (from 4.43 ± 0.55 to 3.89 ± 0.42 *p* < 0.001, from 5.06 ± 1.29 to 3.32 ± 1.52 *p* < 0.001, from 2.34 ± 0.44 to 2.08 ± 0.29 *p* = 0.003, respectively) [26]. In the present intervention, the effect of 4 g/day EPA and DHA supplements on the lipid profile of dyslipidemic patients on stable statin therapy was explored.

## Methods

### Participants

Eligible participants were Caucasian men or women aged between 50 and 79 years who had been receiving a stable dose of atorvastatin for the control of serum LDL-C concentration for at least for 8 weeks before initial screening. Inclusion criteria was current combined hyperlipidemia with a non-HDL-C level above 130 mg/dL as per the National Lipid Association treatment goals [2].

Exclusion criteria included current use of n-3 FA supplements, patients that had any type of heart surgery, patients that were diagnosed with any type of cancer and/or have had any kind of cancer therapy, patients that have had kidney failure and patients that have had liver failure in the past 6 months, as well as ongoing pregnancy or lactation. All patients were asked to provide signed informed consent before study-protocol was conducted.

100 patients who had been prescribed atorvastatin for more than 8 weeks were invited for the initial screening, during which their serum LDL-C was measured. Thirty-eight patients did not meet the inclusion criteria, and 12 eligible patients did not give consent to take part in the intervention. 44 patients, out of the remaining 50, were randomly recruited for the study. The random sampling was carried out using randomizer.com.

### Study design

**Fig. 1:**
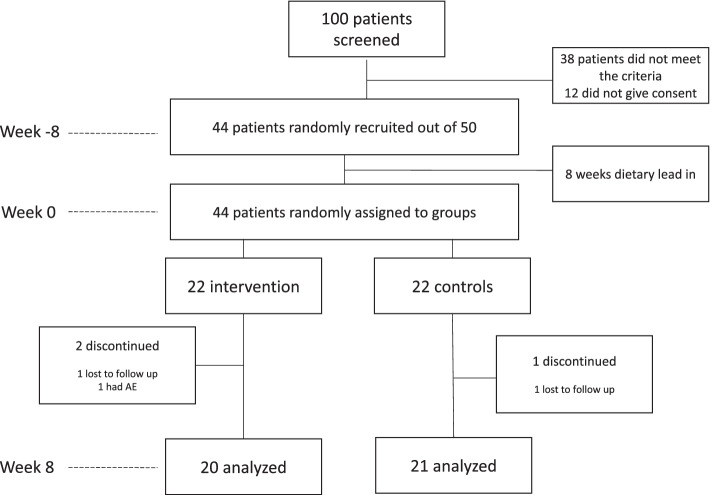
Study flowchart

This was a randomized, double-blind, placebo controlled, parallel groups study. Figure 1 summarizes the trial design. Before entry into the intervention phase of the study, 8-weeks of dietary lead in was done in accordance with NICE guidelines (2014) and patients were instructed to maintain this diet throughout the study. Adherence to dietary advice was measured by a 3-day weighed intake food diary at Week 4. Nutrient content of the diet was estimated using Nutritics software (Nutritics Ltd., Ireland) using UK: SACN 2015 food composition tables. This study was approved by the University of Chester Faculty of Clinical Sciences and Medicine Research Ethics Committee (REF: 1288/17/GD/CSN).

After dietary lead-in, baseline measurements of fasting blood TG, TC, HDL-C, LDL-C, and LDL-C subgroups (LDL I (large buoyant LDL), LDL II (intermediate density LDL), and LDL III (small dense LDL) were carried out for all patients at 2 visits separated by 1 week, and the means were used as baseline values. Non-HDL-C was calculated by subtracting HDL-C from TC. After baseline measurements, all 44 patients were randomized by www.randomizer.com in equal numbers to receive either EPA (75%, 3000 mg) and DHA (25%, 1000 mg) 4 g/day ethyl esters (Wiley’s Finest, USA) or OO (placebo, Ali Raif Pharma, Turkey) 4 g/day for 8 weeks in combination with the same dose of atorvastatin they have been prescribed to. The total EPA and DHA dose recommended by American Heart Association for lipid lowering is approximately 4 g/day [27] which is the common therapeutic dose used in several major clinical trials including COMBOS [17], MARINE [13], ESPIRIT [18], EVOLVE [12], ANCHOR [20]. For all patients, atorvastatin dosage has been kept constant throughout the trial.

Both groups took four 1000 mg capsules orally 4 times daily and compliance was measured by the number of capsules consumed relative to the number estimated to be consumed. n-3 FA tablets included EPA and DHA ethyl esters (1000 g), other n-3 FA (60 mg), fish gelatin, glycerine, purified water and alpha tocopherol, while placebo tablets contained extra virgin olive oil.

Baseline measurements were repeated at the end of week 8. Study participants and investigators remained blinded to all laboratory results until the last subject completed the 8-week intervention period. During the treatment phase, patients attended clinic visits at weeks 4 and 8.

### Biochemical measurements

Laboratory analyses were performed on the serum or serum of 12 hours fasting blood samples. LDL-C, VLDL-C and HDL-C were measured with homogeneous enzymatic colorimetric assay of Roche Diagnostics (USA). TG and TC was measured with enzymatic colorimetric assay of Roche Diagnostics (USA). LDL-C subgroups were analyzed using electrophoresis by Lipoprint (Quantimetrix, USA).

### Statistical analysis

Power analysis was performed by using the G*Power software (v3.1.9) program to determine the sample size. At the baseline, a pilot study was performed with 10 people in each group. When the percentage change in non-HDL values of the groups was investigated, this was found to be 3.25 ± 17.02 in the control group and − 8.86 ± 3.81 in n-3 FA group. According to evaluation performed by using these data, effect size was calculated to be d = 0.982 and each group should include 18 people to obtain 80% power at a level of α = 0.05. Sample size was assigned as 44 to allow for subject attrition and other potential causes of study withdrawal up to 20%. Demographic and baseline analyses were performed for all study participants, whilst efficacy analysis was performed only on patients that successfully completed 8-weeks study protocol. The primary efficacy end point was non-HDL and LDL III particle concentration percentage change from baseline; secondary efficacy end points were changes in TG, TC, LDL-C, VLDL-C and HDL-C.

Normal distribution of the sample was tested with Shapiro-Wilk’s test as sample size was less than 100 [28]. Independent samples t-test was used for the intergroup comparisons of change in quantitative variables from baseline to post-intervention with normal distribution and Mann Whitney U test was used for the intergroup comparisons of quantitative variables without normal distribution. Paired Samples t-test was used for the in-group comparisons of quantitative variables with normal distribution and Wilcoxon Signed Ranks test was used for the in-group comparisons of quantitative variables without normal distribution. Pearson’s chi-square test was used for comparison of qualitative data. Levene’s test was conducted to check homogeneity of variance between groups. *P* < 0.05 was the threshold for statistical significance.

## Results

### Subjects

Of the 44 patients randomly assigned to the trial, 41 have completed it. In the treatment arm, one subject reported adverse effect (nausea) and one was lost to follow-up. In the placebo group one subject was lost to follow-up.

The baseline demographics of the patients are listed in Table 1. The patients were predominantly men (63%) with a mean (±SD) age of 60.62 ± 9.56 years and body mass index of 23.8 ± 3.0 kg/m^2^. There was no statistically significant difference between groups regarding age, BMI values and gender ratios; as well as baseline TG, TC, HDL-C, non-HDL-C, LDL-C, VLDL-C, LDL-C I, LDL II and LDL III concentrations (Tables 1 and 2).

**Table 1:** Baseline characteristics of the participants

		Placebo (***n*** = 21)	n3 FA (***n*** = 20)	***P***
		Mean ± SD	Mean ± SD	
**Age (years)**		60.91 ± 8.56	60.32 ± 9.53	0.830
**BMI (kg/m**^**2**^**)**		24.10 ± 2.94	23.50 ± 3.17	0.525
**Gender, n (%)**	Female	8 (36.4)	8 (36.4)	
	Male	14 (63.6)	14 (63.6)	
**Patients on OADs (%)**		12 (57.1)	10 (50.0)	
**Patients on AHDs (%)**		16 (76.1)	14 (70.0)	

Definitions – *BMI* Body mass index, *OADs* Oral antidiabetic drugs, *AHDs* Antihypertensive drugs

**Table 2:** Comparison of diets during the intervention periods as assessed by dietary records

Dietary Compounds	Placebo	n-3 FA	***P***
	Median (Q_**1**_, Q_**3**_)	Median (Q_**1**_, Q_**3**_)	
**Total energy (kcal)**	3222 (3090, 3420)	3274 (3105, 3420)	0.879
**Total energy (MJ)**	13.5 (12.9, 14.3)	13.7 (13.0, 14.3)	
**CHO (%E)**	51.25 (50.8, 52)	51.25 (50.9, 52)	0.629
***Free Sugars (%E)***	5.25 (5.1, 6.1)	5.25 (5.2, 5.3)	0.820
***Fiber (g)***	18.3 (17.6, 19.4)	17.9 (16.4, 18.8)	0.693
**Protein (%E)**	16.4 (16.3, 17.2)	16.4 (15.9, 17.1)	0.378
**Fat (%E)**	32.1 (31.4, 32.9)	32.1 (30.7, 32.7)	0.859
***SFA (%E)***	9.5 (8.9, 9.9)	9.5 (8.9, 9.8)	0.962
***MUFA (%E)***	10.9 (10.6, 11.3)	11.2 (10.6, 11.9)	0.364
***PUFA (%E)***	9.25 (8.8, 9.6)	9.25 (8.8, 10.8)	0.972
***EPA (%E)***	0.91 (0.89, 0.98)	0.91 (0.89, 0.97)	0.730
***DHA (%E)***	0.92 (0.91, 0.97)	0.92 (0.91, 0.97)	0.392

Values are median (Q_1_, Q_3_), Q_1_: First quartile and Q_3_: Third quartile. Values were derived from food composition tables

### Diet

The analysis of total energy derived from dietary components showed no significant difference between the groups (Table 2). No significant change in body weight was observed throughout the intervention.

### Laboratory measurements

Baseline and week 8 values as well as percentage change from baseline to the end of treatment for the primary and secondary endpoints are shown in Table 3.

**Table 3:** Outcome variables (serum) at baseline and the end of treatment

Parameter (mmol/L)		Placebo (***n*** = 21)	n-3 FA (***n*** = 20)	***P***
		Mean ± SD	Mean ± SD	
**TG**	Baseline	1.61 ± 0.85	1.62 ± 0.60	0.924
	8 week	1.66 ± 0.86	1.29 ± 0.37	0.090
	% Difference	6.20 ± 10.14*	−21.51 ± 12.15***	< 0.001***
**TC**	Baseline	4.97 ± 0.81	4.91 ± 0.72	0.820
	8 week	5.05 ± 0.83	4.55 ± 0.65	0.049*
	% Difference	2.06 ± 2.97**	−6.55 ± 3.59***	< 0.001***
**HDL-C**	Baseline	1.40 ± 0.47	1.34 ± 0.32	0.677
	8 week	1.42 ± 0.49	1.37 ± 0.30	0.716
	% Difference	2.90 ± 6.73*	1.09 ± 6.97	0.403
**Non-HDL-C**	Baseline	3.55 ± 0.60	3.47 ± 0.59	0.999
	8 week	3.62 ± 0.72	3.20 ± 0.54	0.042*
	% Difference	4.65 ± 21.00	−9.47 ± 4.58***	0.007**
**LDL-C**	Baseline	2.61 ± 0.65	2.66 ± 0.61	0.831
	8 week	2.64 ± 0.61	2.64 ± 0.55	0.985
	% Difference	0.91 ± 4.35	−0.08 ± 5.20	0.510
**VLDL-C**	Baseline	0.93 ± 0.25	0.90 ± 0.29	0.684
	8 week	0.98 ± 0.25	0.55 ± 0.20	< 0.001***
	% Difference	3.95 ± 9.93	−36.88 ± 11.75***	< 0.001***
**LDL-C I**	Baseline	1.07 ± 0.35	1.03 ± 0.30	0.689
	8 week	1.08 ± 0.36	1.03 ± 0.24	0.647
	% Difference	1.41 ± 10.21	3.89 ± 15.73	0.551
**LDL II**	Baseline	0.56 ± 0.20	0.63 ± 0.26	0.381
	8 week	0.56 ± 0.19	0.67 ± 0.30	0.209
	% Difference	−0.74 ± 16.24	1.55 ± 17.27	0.664
**LDL III** **median (Q**_**1**_**, Q**_**3**_**)**	Baseline	0.05 (0.02, 0.10)	0.09 (0.05, 0.15)	0.158
	8 week	0.05 (0.02, 0.10)	0.02 (0.00, 0.08)	0.090
	% Difference	0 (0, 0)	−67.5 (−100, −31.25)**	< 0.001***

Q_1_: First quartile, Q_3_: Third quartile *P < 0.05, ** *P* < 0.01, *** *P* < 0.001

*P*-value represents difference between groups at the relevant timepoint for each parameter. *P*-value for % difference represents the effect of intervention for that parameter

### Primary endpoints

For LDL III concentration, the percent change over 8 weeks, in serum LDL III concentration in n-3 FA group was determined to be significantly greater compared to the change in the placebo group (− 67.5% vs − 0%, respectively; *P* < 0.001). This was also the case for serum non-HDL-C. The non-HDL value observed in n-3 FA group was determined to be statistically significantly different compared to the placebo group (− 9.47% vs + 4.65%, respectively; *P* = 0.007).

### Secondary endpoints

**Fig. 2:**
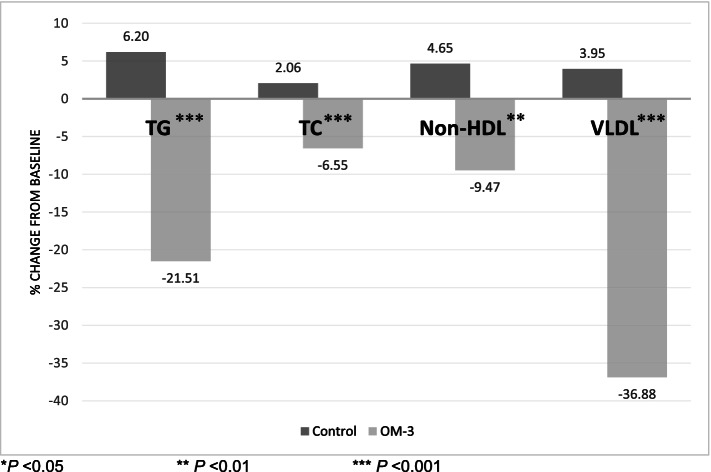
Mean percent change in TG, TC, Non-HDL and VLDL from baseline to the end or treatment. *P < 0.05, ** *P* < 0.01, *** *P* < 0.001

Percent change over 8 weeks, in serum TG (− 21.5% vs 6.2%, *P* < 0.001) and VLDL-C (− 36.9% vs 4.0%, *P* < 0.001) and TC (− 6.6% vs 2.1%, *P* < 0.001) were significantly greater in the n-3 FA group compared to the placebo group respectively (Fig. 2).

## Discussion

In this randomized, parallel-group, double-blind, placebo-controlled trial of men and women with hyperlipidemia on statins, 4 g/d EPA + DHA supplementation produced significant reductions in the primary endpoints of non-HDL-C and LDL-III particle concentration (− 9.47 ± 4.58, *p* < 0.001 and − 67.5 (− 100, − 31.25), *P* < 0.01, respectively) compared with OO.

There are relatively few studies investigating the role of marine n-3 FA in altering LDL-C particle phenotype. In a recent randomized controlled trial (*n* = 53), 4 g/day prescription n-3 FA supplementation for 8 weeks resulted in significant increases in LDL particle sizes and significant decreases in blood lipid, lipoprotein and apolipoprotein concentrations in the intervention arm [29]. An earlier single-blind placebo controlled study that randomized hyperlipidemic subjects (*n* = 33) into three groups (pravastatin, 6 g/day EPA and DHA, placebo) initially for 6 weeks and then provided combined pravastatin and 6 g/day EPA and DHA therapy to all subjects for 12 weeks, reported a significant increase in LDL stokes’ diameter from 25.0 to 25.9 nm (*P* < 0.05) in the n-3 FA group, although LDL III particle concentration was not measured in this study [30]. In another randomized controlled trial (*n* = 42), in which subjects were randomized to atorvastatin alone or atorvastatin and 1.68 g/day EPA and DHA for 5 weeks after dietary run in, a significant reduction in LDL III particle concentration (− 1.23 mmol/L, *P* < 0.05) in the treatment arm was reported [21]. However, this change was not significantly different when compared to the change in the placebo group. Maki et al. found a significant increase in LDL-C particle size from 19.9 (19.2, 22.0) nm to 20.4 (19.3, 21.7) nm (*P* = 0.024) in their cross-over study (*n* = 39) when 4 g/day EPA and DHA supplementation was combined with simvastatin [22]. Different from a previous study [30], Maki et al. also measured LDL III particle concentration and reported no significant change between control and intervention arms. However, in more recent studies, n-3 FA together with statins partially improved both lipoprotein particle size and concentration [25, 26, 29]. It is of particular importance that this present study showed a difference in LDL III particle concentration, given the fact that LDL III and IV particle concentration is suggested to be a stronger predictor of CVD than LDL-C particle size [31, 32].

Previous studies have shown similar results regarding the effect of n-3 FA supplementation on serum non-HDL-C concentrations. The COMBOS trial reported a 9.1% reduction (*P* < 0.001) in non-HDL-C with dietary supplementation of 4 g/day EPA and DHA during 40 mg/day simvastatin therapy compared to 2.2% (*P* < .001) with corn oil [17]. The same dose of n-3 FA and statin caused 6.9% reduction (*P* < 0.01) in ESPIRIT trial, compared to 0.9% (*P* > 0.05) with olive oil [18]. Similarly, in respective studies of combination therapy, approximately 10% significant reduction in non-HDL-C was reported [16, 21, 24, 33]. Higher reductions in have also reported in the past. Maki et al. for instance, showed 40% reduction (*P* < 0.001) in their study of a similar design with 39 patients, however, the control group also showed a large significant reduction in serum non-HDL-C concentrations (34%, *P* < 0.001) [22]. Furthermore, in the ANCHOR trial, a 15% reduction (*P* < 0.0001) in non-HDL-C was also reported [20]. This could be due to the use of EPA ethyl esters alone in this study, given that EPA might lead to greater average reductions in non-HDL-C than DHA [34]. However, little is known about the individual effects of these fatty acids on distinct lipids, and studies suggest they have complementary roles to each other, therefore their combined use is more widely preferred, as in the present study.

In the present study, the decrease in non-HDL-C cholesterol in the n-3 FA group was likely achieved mainly due to lowering of VLDL concentrations (− 36.88 ± 11.75%, *P* < 0.0001), as well as other triglyceride-rich lipoproteins such as chylomicron remnants. The significant reduction in VLDL-C concentration is consistent with the previous evidence [17–20, 22]. In human physiology, increased concentrations of VLDL-C particles positively correlate with increased TG concentrations. TG-reducing effects of n-3 FAs are mediated by transcription of several nuclear receptors that play a key role in lipid metabolism, including PPAR-α, which increases fatty acid oxidation in the liver, adipose, heart and skeletal muscle, as well as sterol regulatory element binding proteins (SREBP), especially SREBP-1c, the major activator of hepatic lipogenesis [11]. PUFA metabolites, such as eicosanoids and oxylipins, are potent activators of PPARs [35]. Through these mechanisms, marine n-3 FA may downregulate VLDL metabolism through decreasing TG synthesis and increasing triglyceride clearance.

The significant 21.51 ± 12.15% (*P* < 0.001) reduction in TG in the present study is slightly lower in magnitude than the findings of some o previous studies. Kastelein et al. reported a 31% reduction (*P* < 0.001) and Bays et al. reported 33% (*P* < 0.0001) reduction in serum TG concentrations with 4 g/day EPA and DHA monotherapy [12, 13]. When the same n-3 FA dose was combined with a statin, a 28% reduction was reported [17]. Similarly, in a randomized controlled trial of hyperlipidemic patients (*n* = 56), co-administration of 4 g/day n-3 FA with statin treatment for 16 weeks reduced serum TG concentrations more effectively than statin monotherapy (− 34.8% vs. -15.2%, *P* = 0.0176) [36]. The relatively smaller reduction in TG achieved in the present study may be due to the difference in the baseline characteristics of the patients when compared to the aforementioned studies. Mean (±SD) baseline serum TG concentrations of the intervention group in the present study (145.05 ± 52.67 mg/dL, 1.62 ± 0.60 mmol/l) were much lower than the EVOLVE and MARINE trials (655 and 679 mg/dL, 7.40 and 7.67 mmol/l, median values, respectively) and the percent reduction in TG concentrations highly depends on the baseline values [37, 38]. This is supported by the mean (±SD) baseline TG concentration of the n-3 FA groups in ESPIRIT trial which was 287 ± 82.8 mg/dL (3.25 ± 0/94 mmol/l) and the study achieved a reduction in serum TG concentration of − 20%, (*P* < 0.01), similar to the present study [18].

Reducing serum TG concentration is important for two reasons. Firstly, TG, as a substrate of LDL-C synthesis, is deemed a target in clinical management of dyslipidemia and each 1 mmol/l reduction in TG concentrations is believed to reduce CVD risk by 14% in men and 37% in women [39]. More importantly, it has been long known that clinically significant reductions in TG concentrations are typically accompanied by a shift in LDL particle size from smaller and denser particles (LDL III, IV) to larger and more buoyant particles (LDL-C I, II) [40]. Serum TG concentration are inversely related to the LDL-C particle size and it can been hypothesized that reducing TG would result in an increase in LDL-C particle size [41]. In the present study, the significant decrease in serum concentration of LDL III (− 67%, *P* < 0.01) from 3.5 mg/dL to 1 mg/dL in the n-3 FA group is likely to be linked to the significant decrease in serum TG.

In the placebo group, serum TC, TG and HDL-C concentrations at week 8 had shown minor significant changes from baseline. One possible explanation for changes in the placebo group in the present study is that late-onset effects of the dietary run-in and continued adherence to the healthy and balanced diet might have improved these variables.

### Strengths and limitations

The main strength of the present study is its prospective, randomized, double-blinded and placebo-controlled design. Furthermore, the study population was carefully determined and there were no baseline differences between control and intervention groups. Additionally, small and dense LDL-C was measured with gel electrophoresis that is a strong method for detecting types of lipoprotein particles.

The present study also has certain limitations. First, the small sample size (although powered) and short intervention duration, limits the conclusions that can be drawn. Although the lipid alterations achieved in this study are consistent with the findings of larger studies with longer duration, findings should still be confirmed in a larger population. Furthermore, the capsule load was high (4 capsules per day), and compliance was measured only by capsule count. Ideally, compliance needs to be assessed by plasma and erythrocytes EPA and DHA content. Indeed, the plasma and erythrocyte fatty acid composition should be measured and compared in any further studies.

Like many other studies, this study tested a supplemental form of n-3 FA containing EPA and DHA in ethyl ester (EE) form. However, n-3 FA supplements are also available in TG form and there is ongoing debate about whether different chemical forms of EPA and DHA are absorbed in an identical way by the human body. Some previous findings suggest a comparable bioavailability, whereas others reported a higher bioavailability from TG. An earlier RCT (*n* = 150) that tested long-term (6 months) moderate consumption (1.68 g/day) of both chemical forms concluded that TG n-FA led to a faster and higher increase in the erythrocyte’s membrane EPA and DHA content [42]. In a more recent trial (*n* = 22), short term bioavailability of the EE and TG, measured by plasma concentrations of EPA, and DHA, did not differ after 24 hours a single oral dose of ∼1.2 g [43]. Taken together, there is limited evidence to compare bioavailability of EE and TG n-3 FA. However, recent studies point out that EPA and DHA in free fatty acids (FFA) form may have 4-fold greater bioavailability than n-3 FA ethyl esters given that their absorption does not involve pancreatic lipase. Particularly when taken on an empty stomach, the FFA formulation may have provided great flexibility in the dosing schedule. Unfortunately, this prescription n-3 FA was not available to the researchers.

The use of OO as a placebo may have had non-neutral effects on the outcome variables due to its high oleic acid content and the role of oleic acid in CVD prevention [44]. However, 4 g/day was deemed too low a dose to bias the result, especially when compared to the amount used in studies such as PREDIMED, which showed that 50 g/day use of OO reduced CVD risk [45], several studies have used OO as placebo given the lack of a true placebo.

The present study included participants with a normal body weight – which was preserved throughout the intervention. In obese subjects, similar results in lipid profile might be observed [46], also accompanied by a reduction in inflammatory markers [47].

Additionally, physical activity was not monitored throughout the trial. Physical activity is an important factor that effects lipid metabolism and ideally should be evaluated in studies looking into blood lipids.

Lastly, although the present studies and others have demonstrated beneficial effects of n-3 FAs in relation risk, it should be noted that dietary pattern, and more generally, lifestyle factors are stronger determinants of CVD risk compared to the effect of single nutrients alone.

## Conclusion

There is a clinical need to effectively reduce serum small, dense LDL-C particle concentration for CVD prevention. Agents that lower serum TG concentrations are usually successful in altering LDL-C particle phenotype. However, the efficacy of n-3 FAs, which are TG lowering agents, in LDL-C particle altering has not been widely investigated. In this double-blind, randomized, controlled trial of combined use of n-3 FA with atorvastatin in patients with hyperlipidemia, supplementation of EPA and DHA at 4 g/d dosage improved the overall lipid profile in 8 weeks, including significant lowering of serum LDL III and non-HDL-C concentrations. Further large-scale studies are required to confirm the results of this trial.

## Data Availability

The datasets used and/or analysed during the current study are available from the corresponding author on reasonable request.

## Abbreviations

CVD: Cardiovascular disease
DHA: Docosahexaenoic acid
EE: Ethyl ester
EPA: Eicosapentaenoic acid
HDL-C: High density lipoprotein cholesterol
LDL-C: Low-density lipoprotein cholesterol
n-3 FA: Omega 3 fatty acids
non-HDL-C: Non-high density lipoprotein cholesterol
OO: Olive oil
PPAR- α: Peroxisome proliferator–activated receptor-α
PPARs: Peroxisome proliferator–activated receptors
SREBP1-c: Sterol responsive element binding protein-1c
TC: Total cholesterol
TG: Triglycerides
VLDL-C: Very-low-density lipoprotein cholesterol
